# Probable REM sleep behavior disorder is associated with longitudinal cortical thinning in Parkinson’s disease

**DOI:** 10.1038/s41531-021-00164-z

**Published:** 2021-03-02

**Authors:** Eun Jin Yoon, Oury Monchi

**Affiliations:** 1 Department of Clinical Neurosciences, University of Calgary, Calgary, AB Canada; 2 Hotchkiss Brain Institute, Cumming School of Medicine, Calgary, AB Canada; 3 Department of Radiology, University of Calgary, Calgary, AB Canada; 4 Centre de Recherche Institut, Universitaire de Gériatrie de Montréal, Montréal, QC Canada

**Keywords:** Parkinson's disease, Parkinson's disease

## Abstract

REM sleep behavior disorder (RBD) has a poor prognostic implication in both motor and non-motor functions in Parkinson’s disease (PD) patients. However, to the best of our knowledge no study to date investigated the longitudinal cerebral changes underlying RBD symptoms in PD. We performed the longitudinal study to investigate the association between probable RBD and cortical and subcortical changes in early, de novo PD patients. We studied 78 participants from the Parkinson’s Progression Marker Initiative who underwent structural MRI at baseline and after 2 years. The presence of probable RBD (pRBD) was evaluated using the RBD screening questionnaire. We compared the cross-sectional and longitudinal cortical thickness and subcortical volume changes, between PD patients with and without pRBD. At baseline, we found bilateral inferior temporal cortex thinning in the PD-pRBD group compared with the PD-noRBD group. Longitudinally, the PD-pRBD group revealed a significant increase in the rate of thinning in the left insula compared with the PD-noRBD group, and the increased thinning correlated with decreased cognitive performance. In subcortical volume analyses, the presence of pRBD was linked with volume decrease over time in the left caudate nucleus, pallidum and amygdala. The volume changes in the left caudate nucleus revealed correlations with global cognition. These results support the idea that RBD is an important marker of rapid progression in PD motor and non-motor symptoms and suggest that the atrophy in the left insula and caudate nucleus might be the underlying neurobiological mechanisms of the poorer prognosis in PD patients with RBD.

## Introduction

Rapid eye movement (REM) sleep behavior disorder (RBD), a parasomnia manifested by vivid dreams associated with simple or complex motor behavior during REM sleep, is one of the most common non-motor symptoms in Parkinson’s disease (PD). While the prevalence of RBD is less than 2% in the general population^1,2^, a recent meta-analysis reported a pooled prevalence of RBD of 42.3% in PD^3^. Moreover, RBD occurs in up to 25% of de novo PD patients^2^ and the prevalence increases with disease progression^4,5^. Increasing evidence has suggested that the presence of RBD in PD patients is associated with severity, and a quicker evolution of motor and non-motor symptoms. PD patients with RBD have worse cognitive performance, higher MCI frequency^6,7^, and higher rate of future development of dementia^8,9^ compared with PD patients without RBD. Also, the presence of RBD in PD is associated with faster motor progression^10,11^, future development of hallucination^9,12^, autonomic dysfunction and impaired color vision^13^.

Based on animal studies, RBD is thought to be associated with the dysfunction of a network of brainstem nuclei^14^. Several cross-sectional neuroimaging studies in PD patients with RBD found structural changes in the brainstem regions, such as reduced signal intensity in the locus coeruleus/subcoeruleus, using neuromelanin-sensitive imaging^15^ and volume contraction in the pontomesencephalic tegmentum and medullar reticular formation, using deformation-based morphometry (DBM)^16^. However, studies using diffusion tensor imaging found no differences in brainstem structures in PD patients with RBD compared to those without RBD^17,18^. Moreover, in a postmortem study of patients with Lewy body disease, there were no differences in either degree of neuronal loss or burden of α-synuclein pathology in the pontine tegmentum, regardless of RBD symptoms during life, suggesting that pathology in the brainstem alone may not be sufficient to cause RBD in PD^19^.

Although the previous studies have suggested the importance of RBD as a prognostic factor of rapid progression in PD motor and non-motor symptoms and possible mechanisms of RBD in PD patients beyond brainstem structures, little is known about the cerebral changes associated with RBD in PD. Moreover, to our knowledge, no study to date has investigated the effect of the early presence of RBD on the pattern of brain atrophy overtime in PD. As a sensitive marker of gray matter atrophy, cortical thinning has been associated with idiopathic RBD^20,21^, PD with concomitant RBD^22^, and a risk of conversion to a Lewy body disorder in idiopathic RBD patients^23^. Therefore, in this study, we investigated the association between RBD and changes of cortical thickness and subcortical volumes in early, de novo PD patients from the Parkinson’s Progression Markers Initiative (PPMI) project^24^ (http://www.ppmi-info.org), cross-sectionally and longitudinally. We hypothesized that PD patients with RBD would reveal increased brain atrophy compared to those without RBD, cross-sectionally and longitudinally and that these changes would correlate with their motor and non-motor symptoms.

## Results

The demographic and clinical characteristics of the participants included in this study can be found in Table 1. Eighteen PD-pRBD patients and sixty PD-noRBD patients were included in this study. Most of the participants received dopaminergic treatment during follow-up (94% of PD-pRBD and 84% of PD-noRBD; no group difference, Fisher’s exact test, *p* = 0.441). Part of the participants received medications that can affect RBD symptoms, including antidepressants^25,26^, beta blockers^26^, clonazepam, and melatonin. However, there were no significant group differences in the proportions of the use of those medications (Table 1). The PD-pRBD group revealed significant higher SCOPA-AUT scores at baseline and longitudinal increase compared to the PD-noRBD group. There were no group differences in baseline cognitive scores and longitudinal changes of those scores. At baseline, depression and anxiety symptoms in the PD-pRBD were not statistically different from the PD-noRBD group, but longitudinally, the PD-pRBD group revealed increased STAI scores compared with the PD-noRBD group.

**Table 1:** Demographic and clinical data.

	PD with pRBD (*n* = 18)	PD without RBD (*n* = 60)	*p*-value^a^
Age at baseline, mean ± SD (range), years	64.9 ± 7.9 (52.8–82.3)	62.3 ± 7.2 (50.5–77.3)	0.321
Female, No (%)	2 (11.1)	26 (43.3)	0.013
Education, median ± IQR (range), years^b^	16.0 ± 4.0 (10.0–20.0)	16.0 ± 5.0 (8.0–22.0)	0.847
Months since diagnosis at baseline, median ± IQR (range)	3.0 ± 8.5 (0.4–23.7)	4.1 ± 7.2 (0.7–35.8)	0.236
MRI interval, median ± IQR (range), years	2.03 ± 0.17 (1.96–1.49)	2.01 ± 0.10 (1.75–2.34)	0.099
Accumulated LED, median ± IQR (range)^c^	152,062.5 ± 115,242.7 (0.0–385,000)	118,370.0 ± 170,822.8 (0.0–461,250.0)	0.174
Medication, No (%) at baseline/time 2^b^			
Antidepressant	6 (33.3)/7 (38.9)	13 (21.7)/16 (26.7)	0.354/0.566
Beta blocker	5 (27.8)/4 (22.2)	7 (11.7)/8 (13.3)	0.134/0.457
Clonazepam	1 (5.6)/1 (5.6)	0 (0.0)/0 (0.0)	0.231/0.231
Melatonin	1 (11.1)/2 (11.1)	1 (1.7)/1 (1.7)	0.411/0.131
RBDSQ at baseline, median ± IQR (range)	8.0 ± 2.3 (6.0–12.0)	3.0 ± 3.0 (1.0–5.0)	<0.001
UPDRS-III			
Baseline, median ± IQR (range)	19.0 ± 24 (6.0–38.0)	18.0 ± 11.5 (4.0–47.0)	0.660
Change, median ± IQR (range)	0.4 ± 8.7 (−8.8–22.6)	1.0 ± 5.7 (−7.5–12.0)	0.415
SCOPA-AUT			
Baseline, median ± IQR (range)	11.0 ± 7.0 (6.0–27.0)	6.0 ± 5.0 (1.0–22.0)	0.002
Change, mean ± SD (range)	2.0 ± 1.7 (−0.9–4.8)	0.9 ± 1.9 (−3.6–5.6)	0.039
Montreal cognitive assessment			
Baseline, median ± IQR (range)	27.0 ± 3.3 (20.0–29.0)	28.0 ± 2.8 (21.0–30.0)	0.142
Change, median ± IQR (range)	−0.5 ± 2.1 (−3.2–2.0)	−0.5 ± 1.5 (−4.3–2.5)	0.454
HVLT-R retention			
Baseline, mean ± SD (range)	46.9 ± 7.8 (31.0–56.0)	45.9 ± 13.2 (20.0–67.0)	0.695
Change, mean ± SD (range)	0.6 ± 8.1 (−11.7–15.2)	1.6 ± 7.3 (−13.2–22.3)	0.599
HVLT-R recognition discrimination index			
Baseline, mean ± SD (range)	27 ± 3.25 (20–29)	28 ± 2.75 (21–30)	0.112
Change, mean ± SD (range)	0.7 ± 9.8 (−18.6–17.4)	1.6 ± 6.4 (−15.3–17.5)	0.712
Semantic fluency			
Baseline, mean ± SD (range)	48.2 ± 10.8 (22.0–66.0)	52.7 ± 9.0 (35.0–76.0)	0.077
Change, mean ± SD (range)	−0.8 ± 4.9 (−8.7–10.4)	0.9 ± 4.2 (−7.5–10.8)	0.195
Letter number sequencing			
Baseline, mean ± SD (range)	10.5 ± 3.3 (4.0–15.0)	11.7 ± 2.7 (5.0–19.0)	0.132
Change, mean ± SD (range)	−0.5 ± 1.2 (−2.6–2.0)	−0.1 ± 1.5 (−4.0–3.2)	0.277
Benton judgment of line orientation			
Baseline, mean ± SD (range)	11.5 ± 3.1 (4.5–15.5)	12.4 ± 2.9 (5.7–16.8)	0.250
Change, mean ± SD (range)	0.4 ± 1.4 (−2.3–3.6)	0.0 ± 1.6 (−5.7–4.0)	0.347
Symbol digit modalities			
Baseline, mean ± SD (range)	42.1 ± 11.1 (22.5–58.8)	45.3 ± 7.0 (30.0–63.0)	0.269
Change, mean ± SD (range)	−1.5 ± 3.8 (−8.9–6.4)	0.6 ± 4.4 (−9.8–13.8)	0.068
Geriatric depression scale			
Baseline, median ± IQR (range)	2.0 ± 1.0 (0.0–7.0)	1.0 ± 3.0 (0.0–13.0)	0.660
Change, median ± IQR (range)	0.5 ± 1.4 (−1.0–7.5)	0.0 ± 0.8 (−2.5–7.1)	0.238
State-trait anxiety inventory			
Baseline, median ± IQR (range)	64.5 ± 16.0 (45.0–108.0)	57.0 ± 23.0 (40.0–108.0)	0.087
Change, median ± IQR (range)	4.7 ± 9.9 (−24.6–14.9)	0.2 ± 5.0 (−12.1–7.9)	0.017

*RBD* rapid eye movement sleep behavior disorder, *pRBD* probable RBD; *LED* levodopa equivalent dose; *RBDSQ* RBD screening questionnaire, *UPDRS-III* motor section of the unified Parkinson’s disease rating scale, *SCOPA-AUT* Scales for Outcomes in PD-Autonomic dysfunction, *HVLT-R* Hopkins verbal learning test-revised, *SD* standard deviation, *IQR* interquartile range.

^a^Variables with mean ± SD compared via independent t-test and variables with median ± IQR compared via Mann–Whitney U test.

^b^Fisher’s exact test was used for the comparisons of the binary variables.

^c^Accumulated LED during the follow-up year was calculated for each patient^47^.

### Cortical thinning at baseline

**Fig. 1:**
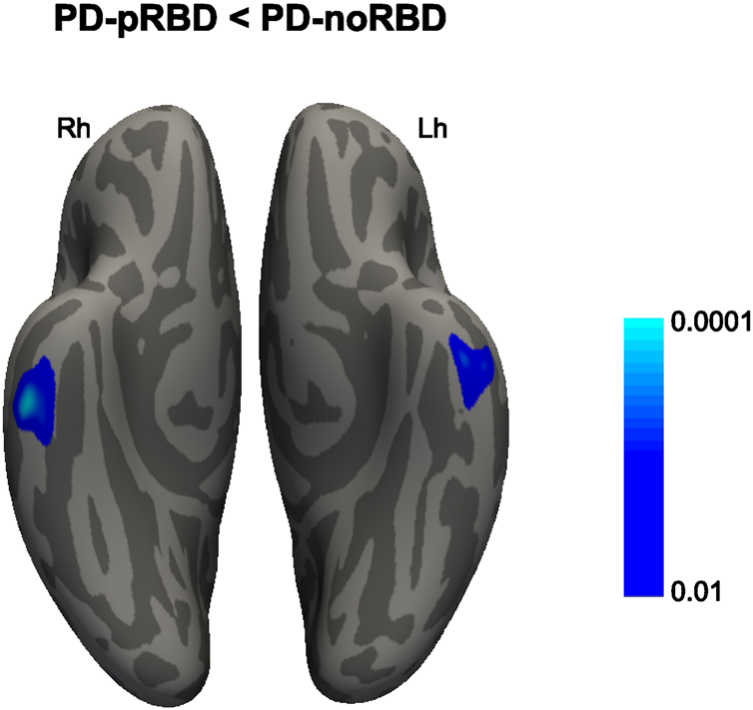
Cross-sectional cortical thickness differences. PD patients with probable RBD (PD-pRBD) revealed a significant thinner cortex in the bilateral inferior temporal cortex compared with the PD patients without RBD (PD-noRBD). The color scale bar shows the *p*-values. Lh, left hemisphere; Rh, right hemisphere.

The PD-pRBD group revealed a significant thinner cortex in the bilateral inferior temporal cortex compared with the PD-noRBD group (MNI coordinates *x* = −48, *y* = −13, *z* = −39, cluster size = 351 mm^3^, cluster-wise *p* = 0.044; *x* = 57, *y* = −20, *z* = −32, cluster size = 512 mm^3^, cluster-wise *p* = 0.005; Fig. 1). There were no group differences in volumes of subcortical structures (Table 2). We did not find significant correlations between the mean thickness of the inferior temporal cortex and clinical variables at baseline.

**Table 2:** Subcortical volume.

	PD with pRBD		PD without RBD		*p*-value^a^	
	Left	Right	Left	Right	Left	Right
Amygdala						
Baseline^b^	1009.7 ± 158.8	1087.9 ± 115.5	1044.7 ± 137.7	1124.1 ± 128.2	0.537	0.537
SPC (*p*-value^c^)	−1.87 ± 2.56 (0.016)	−0.61 ± 1.23 (0.057)	0.07 ± 2.10 (0.917)	−0.47 ± 1.65 (0.051)	0.028	0.081
Caudate nucleus						
Baseline	2233.4 ± 290.3	2258.3 ± 271.6	2172.3 ± 287.1	2235.9 ± 221.1	0.537	0.657
SPC (*p*-value)	−1.73 ± 1.97 (0.014)	−0.96 ± 1.36 (0.016)	−0.79 ± 1.11 (<0.001)	−0.75 ± 1.27 (<0.001)	0.028	0.552
Hippocampus						
Baseline	2496.2 ± 291.2	2566.1 ± 306.8	2584.0 ± 272.8	2653.5 ± 292.4	0.537	0.537
SPC (*p*-value)	−1.17 ± 1.41 (0.014)	−1.05 ± 1.44 (0.016)	−0.49 ± 1.13 (0.004)	−0.67 ± 1.34 (0.001)	0.186	0.408
Nucleus accumbens						
Baseline	312.7 ± 62.3	336.9 ± 59.6	322.6 ± 63.4	331.3 ± 64.1	0.877	0.537
SPC (*p*-value)	−0.85 ± 4.15 (0.425)	−2.06 ± 3.00 (0.016)	0.08 ± 5.89 (0.425)	−0.08 ± 2.95 (0.917)	0.834	0.313
Pallidum						
Baseline	1287.7 ± 134.7	1208.7 ± 124.1	1323.8 ± 136.1	1281.1 ± 141.3	0.537	0.537
SPC (*p*-value)	−1.47 ± 2.10 (0.016)	−0.04 ± 1.52 (0.915)	−0.03 ± 1.72 (0.917)	0.35 ± 1.41 (0.081)	0.028	0.531
Putamen						
Baseline	2838.2 ± 354.8	2855.1 ± 289.8	2989.1 ± 317.9	2961.4 ± 284.9	0.537	0.537
SPC (*p*-value)	−1.09 ± 1.64 (0.019)	−1.16 ± 1.89 (0.025)	−0.54 ± 1.35 (0.005)	−0.53 ± 1.22 (0.002)	0.186	0.186
Thalamus						
Baseline	4311.5 ± 435.8	4302.6 ± 410.9	4500.0 ± 413.2	4415.1 ± 410.7	0.537	0.877
SPC (*p*-value)	−1.22 ± 1.36 (0.014)	−0.84 ± 1.45 (0.032)	0.57 ± 1.15 (0.001)	−0.51 ± 1.45 (0.002)	0.134	0.531

*RBD* rapid eye movement sleep behavior disorder; *pRBD* probable RBD; *SPC* symmetrized percent change.

Values are expressed as mean ± standard deviation.

^a^False discovery rate (FDR)-adjusted *p*-value.

^b^Estimated total intracranial volume normalized values.

^c^FDR-adjusted *p*-value for one-sample t-test.

### Longitudinal changes in cortical thickness and subcortical volume

**Fig. 2:**
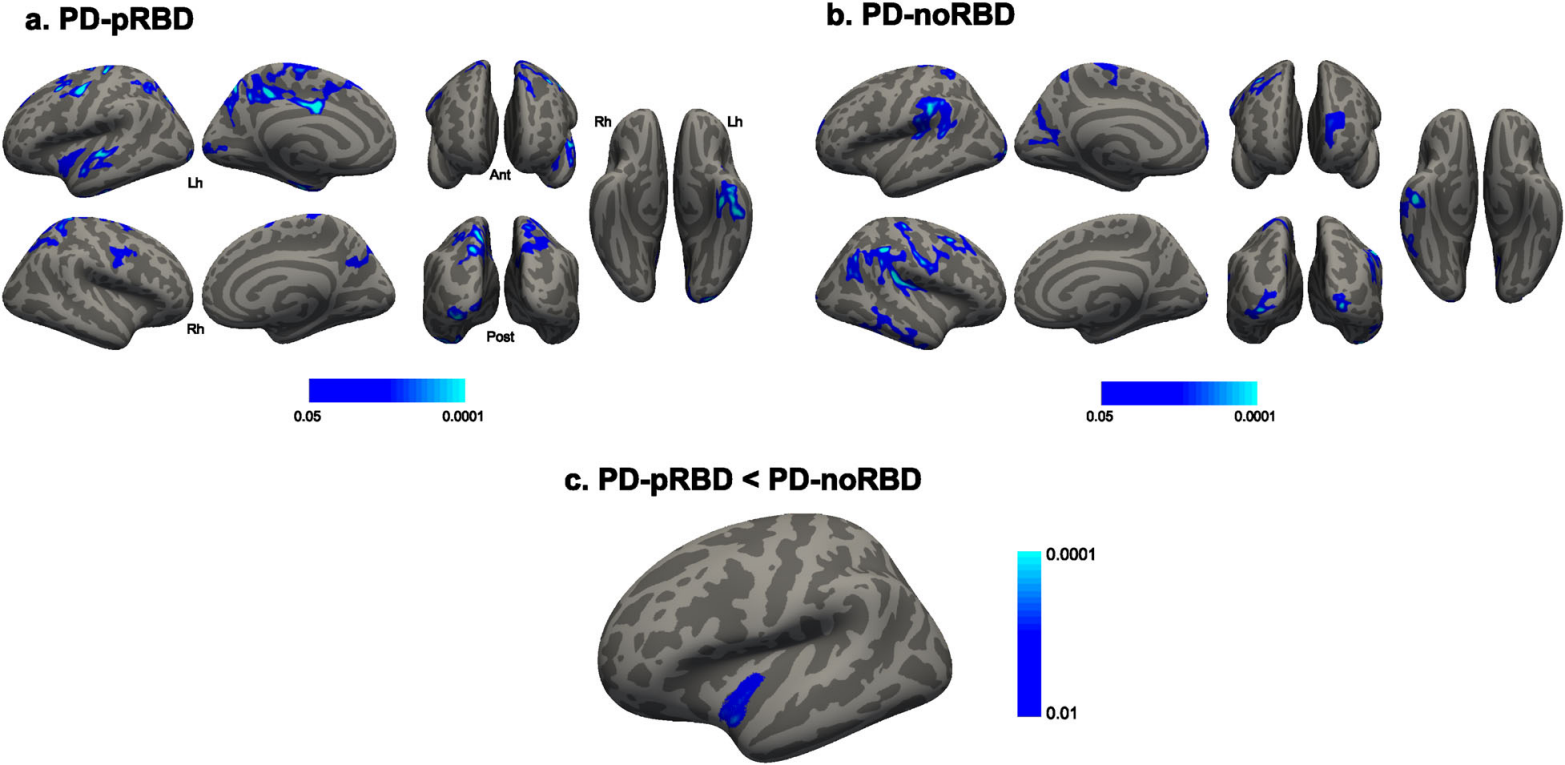
Longitudinal changes in cortical thickness. The symmetrized percent change (SPC) over 2 years of cortical thickness in (**a**) PD patients with probable RBD (PD-pRBD), (**b**) PD patients without RBD (PD-noRBD), (**c**) and the difference between two groups. The PD-pRBD group revealed a significantly increased rate of cortical thinning in the left insular cortex compared with the PD-noRBD group. The color scale bars show the *p*-values. Lh, left hemisphere; Rh, right hemisphere; Ant, anterior; Post, posterior.

The maps of vertex-wise SPC in each group is shown in Fig. 2. The PD-pRBD group showed cortical thinning over time in the precentral and superior parietal cortex bilaterally. The left hemisphere revealed more extend cortical thinning over time in the inferior and superior temporal cortex, insula, precuneus, lateral occipital cortex, caudal middle frontal cortex, and posterior cingulate cortex (Fig. 2a). In the PD-noRBD group, the bilateral precentral, supramarginal and lateral occipital cortex showed cortical thinning over time. In addition, cortical thinning over time in the left superior parietal, cuneus, superior frontal cortex and the right inferior temporal, caudal middle frontal and inferior parietal cortex was found (Fig. 2b). When compared to PD-noRBD group, the PD-pRBD group revealed a significantly increased rate of cortical thinning in the left insular cortex, compared with the PD-noRBD group (MNI coordinates *x* = −41, *y* = −5, *z* = −20, cluster size = 422 mm^3^, cluster-wise *p* = 0.0001; Fig. 2c).

Regarding subcortical volumes, the PD-pRBD group revealed significantly decreased volumes over time in all subcortical structures bilaterally except the left nucleus accumbens and right amygdala and pallidum. The PD-noRBD group also showed decreased volumes over time in the bilateral caudate nucleus, hippocampus and putamen, and right thalamus, while the right pallidum and thalamus revealed relatively increased volume over time. In the group comparison analyses, the longitudinal decrease of the left caudate nucleus, pallidum and amygdala volumes in the PD-pRBD group was significantly greater than in the PD-noRBD group (Table 2).

**Fig. 3:**
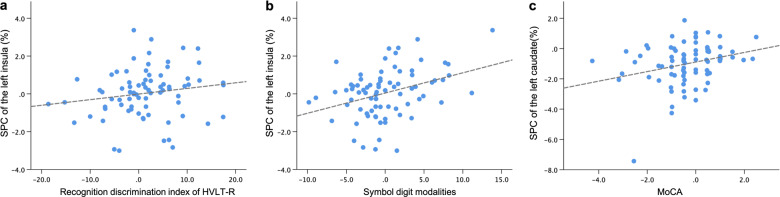
Correlations between SPC and changes in clinical parameters. The mean symmetrized percent changes (SPC) of the left insula positively correlated with (**a**) changes in recognition discrimination index of HVLT-R and (**b**) those in symbol digit modalities. **c** The mean SPC of the left caudate revealed positive correlation with changes in MoCA. The x-axis represents changes in each clinical parameter calculated as: (score at time 2 – score at time 1)/(time 2 – time 1). MoCA, Montreal cognitive assessment; HVLT-R, Hopkins verbal learning test-revised.

The SPC of the significant clusters revealed correlations with clinical variables. The SPC values of left insula cortex positively correlated with changes in HVLT-R recognition discrimination index (rho = 0.310, *p* = 0.008) and symbol digit modalities test (rho = 0.309, *p* = 0.008). Also, the SPC values of the left caudate nucleus positively correlated with changes in MoCA (rho = 0.280, *p* = 0.017) (Fig. 3).

## Discussion

This study investigated the associations between RBD and brain changes in PD cross-sectionally and longitudinally. At baseline, we found bilateral inferior temporal cortex thinning in the PD with pRBD group compared with the PD-noRBD group. Longitudinally, the left insular cortex revealed a significant increase rate of thinning in the PD-pRBD group compared with the PD-noRBD group. Moreover, the increased thinning in the anterior insula correlated with decreased cognitive performances over time. Regarding subcortical volumes, probable RBD was associated with volume decrease in the left caudate nucleus, pallidum and amygdala, over time. The volume changes in the left caudate nucleus revealed a correlation with MoCA score changes.

There are only a few cross-sectional studies of gray matter volume changes associated with RBD symptoms in PD. Previous voxel-based morphometry (VBM) studies have consistently reported smaller volumes in the posterior part of the brain, including temporal and parietal lobe in PD patients with polysomnography (PSG) confirmed RBD^18,22,27^ or those with pRBD^17^ compared with PD patients without RBD. One recent study of surface-based cortical thickness changes found significant cortical thinning in the inferior and superior temporal cortex in PD patients with RBD compared with those without RBD^22^. Our results of bilateral cortical thinning in the bilateral inferior temporal cortex are in line with those previous studies, supporting the importance of the temporal lobe in RBD symptoms even in early, de novo PD.

In terms of the subcortical brain regions, previous studies of PD patients with RBD reported different results, depending on RBD evaluation methods and analysis techniques. VBM studies with PD patients with PSG confirmed RBD reported volume decrease in the hippocampus^18^ or thalamus^28^. However, when using vertex-based shape analysis, PD patients with PSG confirmed RBD reported shape contraction in the putamen^22^. On the other hand, a study using DBM^16^ found smaller putamen, amygdala and thalamus volumes in de novo PD patients with pRBD, compared with those without RBD. In this study, the PD-pRBD group revealed no differences in the subcortical volumes compared with the PD-noRBD group at baseline. Several factors might explain the discrepancy between our findings and previous studies. In particular, the early stage of our PD patients might limit the detection of subcortical volume differences between PD subgroups. A VBM study of early PD patients found no significant differences in subcortical volumes according to the presence of RBD^17^. The techniques used to detect volume changes would also be an important factor of the discrepancy. A previous cross-sectional study using PPMI data found significant volume changes in the subcortical areas in PD patients with pRBD compared with those without RBD using DBM^16^. DBM allows a detection of volume differences in both gray and white matter.

Longitudinally, we found a higher rate of cortical thinning in the left insular cortex in the PD-pRBD group compared with the PD-noRBD group. The insula is considered an integrating hub, linking a variety of functions including sensorimotor processing, social-emotional function, interoceptive function and higher-level cognitive processes^29^. According to Braak’s staging hypothesis of PD progression, the insula is one of the first and most affected cortical regions by alpha-synuclein deposition^30^. A meta-analysis of functional neuroimaging studies in PD found that both the anterior and posterior insula is involved in non-motor functions, including cognitive and affective/behavior, and motor symptoms^31^. In these respects, it is suggested that dysfunction of the insula plays a crucial role in non-motor symptoms of PD, as well as somatosensory dysfunction^32^. Moreover, atrophy in the left insula was found in PD patients with mild cognitive impairment (MCI) compared to those with normal cognition, and the atrophy positively correlated with executive-attention deficits^33^. In RBD patients without neurologic disorders, the presence of MCI was associated with cortical thinning in the bilateral temporal cortex including the insula, and low performance of learning/memory and visuospatial function was correlated to the thinning of these brain regions^34^. Similar to these previous studies, we found significant correlations between the higher rate of the left insula thinning and decline in cognitive performances over time, including memory and cognitive processing speed. On the other hand, previous studies found that RBD is one of the critical determinants of a diffuse malignant subtype of PD based on a comprehensive spectrum of motor and non-motor symptoms. Despite similar age and disease duration, PD patients with the diffuse malignant subtype showed a more rapid progression in overall prognosis, with greater decline in cognition and other non-motor symptoms, as well as motor symptoms compared to those with other subtypes^35^. Together, we suggest that the fast rate of the insula thinning in PD patients with pRBD might be an important neuroimaging predictor of poorer prognosis in PD, particularly cognitive decline.

Although there were no subcortical volume differences at baseline, we found significant longitudinal reduction of the volume in the left caudate, pallidum and amygdala in the PD-pRBD group compared with the PD-noRBD group. In a previous neuroimaging study, idiopathic RBD patients revealed contraction in the left pallidum and the shape contraction correlated with their motor performance^20^. In a longitudinal analysis of PD patients, patients with MCI showed volume loss over time in the amygdala compared to those without MCI, and MoCA score changes over time were associated with the volume reduction^36^. Also, in this study, the increased atrophy rate in the left caudate nucleus revealed correlation with global cognitive decline, assessed by MoCA score. We suggest that the accelerated volume decrease in subcortical areas in PD patients with pRBD might be associated with their poorer motor and non-motor symptoms compared with those without RBD. Moreover, the insula is highly interconnected with the basal ganglia and amygdala anatomically and functionally^37–39^. Future neuroimaging studies of brain connectivity of the insula and its association with motor and non-motor symptoms in PD patients with RBD could clarify the underlying mechanisms of RBD as a predictor of poor prognosis in PD.

There are some limitations to this study. First, RBD was not confirmed by PSG. Although PSG is the gold-standard for diagnosing RBD, RBDSQ has shown good internal consistency in PD patients and good validity for screening of RBD in this population, with a recommended cut-off of 6^40–42^. In this study, we used both the baseline and 2-year follow-up RBDSQ scores and excluded participants if they were classified into a different RBD category at each time point. Therefore, we believe that the longitudinal evaluation of RBDSQ reduces the possibility of misclassification. Second, the PD-pRBD group is relatively small, which might affect the statistical power to detect subtle changes in brain atrophy and non-motor symptom changes. Future longitudinal neuroimaging studies with large cohorts of PD patients with PSG confirmed RBD will be needed.

In summary, RBD in de novo PD patients is associated with cortical thinning in the bilateral inferior temporal cortex cross-sectionally and increased rate of atrophy in the left insula, caudate nucleus, pallidum and amygdala longitudinally. Moreover, the increased atrophy over time correlated with cognitive decline in PD. These results support the idea that RBD is a significant prognostic factor of poor evolution in PD^35,43^ and suggest that the atrophy in the cortical and subcortical regions, particularly the left insula and caudate, might be the underlying neurobiological mechanisms of the worse prognosis in PD patients with RBD.

## Methods

### Participants

The PPMI is a longitudinal multi-site clinical study of de novo individuals with early idiopathic PD^24^. PD subjects in the study are required to be untreated for PD and have either asymmetric resting tremor or asymmetric bradykinesia, or a combination of two signs of PD, including bradykinesia, resting tremor and rigidity. PD diagnosis was confirmed with dopamine transporter imaging using single-photon emission computed tomography. For this study, we included PD patients older than age 50 at baseline, with 3T MRI data and RBD screening questionnaire (RBDSQ) at both the initial visit and 2-year follow-up. Probable RBD was screened on the basis of the RBDSQ with a cut-off score of 6^40^. PD patients with RBDSQ score ≥6 were considered as probable RBD (PD-pRBD) and those with RBD score <6 were considered as without RBD (PD-noRBD). To avoid possible misclassification, only participants categorized in the same RBD group at both time points were included in this study. A total of 111 PD patients had the RBDSQ and 3T MRI data at both time points. Among them, 26 participants had a different RBD category between each time point. Each participating PPMI site received approval from their local institutional review board and obtained written informed consent from all subjects. This study was approved by the Conjoint Health Research Ethics Board at the University of Calgary.

### MRI acquisition and preprocessing

T1-weighted MRI scans were acquired in the sagittal plane on a Siemens MRI scanner at each study site, using a magnetization-prepared rapid-acquisition gradient echo sequence (MPRAGE) (full study protocol: http://www.ppmi-info.org/study-design/research-documents-and-sops/). Images using a non-MPRAGE sequence, such as Spoiled Gradient Echo (SPGR) or a non-Siemens MRI scanner, were excluded in this study.

Each participant’s T1-weighted images were first processed using the FreeSurfer imaging analysis suite (http://surfer.nmr.mgh.harvard.edu/; version 6.0.0). The details of these procedures have been extensively described in prior publications^44,45^. Briefly, the procedure includes skull stripping, registration to Talairach space, segmentation of subcortical white and gray matter structures, intensity normalization, tessellation of the gray/white matter boundaries, automated topology correction, and surface deformation following intensity gradients to optimally place the gray/white (white matter surface) and gray/CSF (pial surface) borders that most accurately define the transition to the other tissue class. Segmented volumes were visually inspected, and the appropriate manual corrections were performed. Next, images were automatically processed with the longitudinal stream in FreeSurfer, where a within-subject template was created, which allows equal treatment of all input images, thus limiting processing bias associated with the use of a particular time-point as the reference image^46^. After processing, the longitudinal data was visually compared to ensure corresponding alignment between the two scans. The subcortical structures including bilateral caudate nucleus, putamen, pallidum, nucleus accumbens, hippocampus, amygdala, and thalamus were also segmented in order to obtain their volumes. Seven of 85 PD patients were excluded from the following statistical analyses because of the low quality of FreeSurfer output.

Changes in cortical thickness were measured in terms of symmetrized percent change (SPC), a robust measure recommended by FreeSurfer developers: 100 × [(Thickness at time 2 – Thickness at time 1)/(time 2 – time 1)]/[0.5 × (Thickness at time 1 + Thickness at time 2)]. The cross-sectional and SPC data was smoothed on the surface with a 10-mm FWHM Gaussian kernel.

### Clinical evaluations

To evaluate the severity of PD motor symptoms, we used the Movement Disorder Society-sponsored revision of the unified PD rating scale (MDS-UPDRS) III score. Scales for Outcomes in PD-Autonomic dysfunction (SCOPA-AUT) were used to evaluate autonomic symptoms. Cognitive assessments included the Montreal cognitive assessment (MoCA; global cognition), the retention and recognition discrimination index of the Hopkins verbal learning test-revised (HVLT-R; memory), semantic fluency and letter number sequencing (executive function), the Benton judgment of line orientation (visuospatial function), the symbol digit modalities test (cognitive processing speed). Depression, and anxiety were assessed using the geriatric depression scale-15 and state-trait anxiety inventory (STAI), respectively. Longitudinal changes were calculated for all clinical scores as:

$$\left( {{\mathrm{Score}}{\kern 1pt} {\mathrm{at}}{\kern 1pt} {\mathrm{time}}{\kern 1pt} 2-{\mathrm{Score}}{\kern 1pt} {\mathrm{at}}{\kern 1pt} {\mathrm{time}}{\kern 1pt} 1} \right)/\left( {{\mathrm{time}}{\kern 1pt} 2-{\mathrm{time}}{\kern 1pt} 1} \right).$$

### Statistical analysis

The differences in demographical and clinical data between the two groups were analyzed using independent t-test, Mann–Whitney U test and Fisher exact test as appropriate.

First, as an exploratory analysis, one-sample t-tests for each group were performed to test whether the cortical and subcortical SPC was significantly different from zero. Correction for multiple comparisons was ensured by cluster-wise correction using Monte Carlo simulation with 10,000 (cluster-wise probability = 0.05, α = 0.05) for the vertex-wise analysis and false discovery rate (FDR) for the analysis of subcortical structures.

Then, vertex-by-vertex group comparisons in cortical thickness were assessed both cross-sectionally (at baseline) and longitudinally (SPC), using a general linear model. Age, gender and education were included as covariates for all comparisons. In all imaging analyses, cluster-wise correction using Monte Carlo simulation with 10,000 iterations was applied (cluster-wise probability = 0.01, α = 0.05). Volumes of subcortical structures were analyzed in the same manner as the vertex-by-vertex analysis using a general linear model. The estimated total intracranial volume was included as an additional covariate for the cross-sectional volume analysis. Results were considered as statistically significant when surviving the *p* < 0.05, corrected for multiple comparisons using FDR.

Mean cortical thickness or SPC values at the identified clusters were computed for each participant to perform further correlation analyses with clinical parameters using Spearman’s partial correlation rho while controlling for age, gender, and education.

### Reporting summary

Further information on research design is available in the Nature Research Reporting Summary linked to this article.

## Supplementary information

Reporting summary

## Data Availability

The data underlying this study are third party data. Interested researchers may apply for access to these data at the following link: http://www.ppmi-info.org/access-data-specimens/download-data/.

## Code availability

The cortical thickness and subcortical volume were analyzed using standard processing pipelines in FreeSurfer (http://surfer.nmr.mgh.harvard.edu/; version 6.0.0). The code corresponding to this manuscript is available upon request to the corresponding author.
